# Supplementary material for: Comparing Survival after Resection of Pancreatic Cancer with and without Pancreatic Cysts: Nationwide Registry-Based Study
Source: Cancers (Basel). 2022 Aug 30;14(17):4228. doi: 10.3390/cancers14174228 (PMC9454588; doi:10.3390/cancers14174228)
Supplement: Supplementary file 1 [file cancers-14-04228-s001.zip › cancers-1857798-supplementary.pdf]

Supplementary table S1. Detailed tumor characteristics

	PCN-PDAC *n* = 233	PDAC *n* = 1761	p-value
Diagnoses per year, ***n*** (%)			N/A
2013	34 (10)	303 (90)	
2014	29 (10)	272 (90)	
2015	34 (10)	308 (90)	
2016	44 (13)	289 (87)	
2017	54 (16)	289 (84)	
2018	38 (11)	300 (89)	
Type of surgery, ***n*** (%)			0.002[a]
PPPD	174 (75)	1464 (83)	
Central	-	3 (.2)	
Distal	47 (20)	262 (15)	
Total	12 (5)	32 (2)	
Differentiation grade[b], ***n*** (%)			0.33
Well differentiated	31 (13)	213 (12)	
Moderately differentiated	138 (59)	980 (56)	
Poorly differentiated	64 (27)	568 (32)	
pTNM stage[c], ***n*** (%)			< 0.001
1A	35 (15)	66 (4)	
1B	29 (12)	122 (7)	
2A	32 (14)	245 (14)	
2B	107 (46)	1006 (57)	
3	25 (11)	262 (15)	
4	5 (2)	60 (3)	

Percentages might not sum to 100% because of rounding.

Abbreviations: CI = confidence interval. N = number. PCN = pancreatic cystic neoplasms. PDAC = pancreatic ductal adenocarcinoma. PPPD = pylorus preserving pancreatoduodenectomy. pTNM = pathological tumor-node-metastasis (TNM) Classification of Malignant Tumors.

[a] p-values were based on Fisher's exact test.

[b] Differentiation grade was missing for 271 (14%) of the patients. Missing data were handled by multiple imputation.

[c] Depending on the time of registration, tumor and lymph node staging were based on either the 7th or 8th edition of the pathological tumor-node-metastasis (TNM) classification of malignant tumors.
